# Supplementary material for: Revealing mechanisms of infectious disease spread through empirical contact networks
Source: PLoS Comput Biol. 2021 Dec 20;17(12):e1009604. doi: 10.1371/journal.pcbi.1009604 (PMC8758098; doi:10.1371/journal.pcbi.1009604)
Supplement: Attachment — Submitted filename: cover_letter_INoDS_revision.pdf [file pcbi.1009604.s007.pdf]

We would like to submit our revised manuscript titled “Revealing transmission mechanisms of infectious diseases through empirical contact networks”.

Based on the first comment from Reviewer # 2, we have updated the layout of Figure 2A. The second comment suggests adding additional replications to our analysis. Unfortunately, this will not be possible for us at this time. Our method is computationally expensive, and the time and resources are unavailable (partially because of the pandemic) for us to complete additional runs. We do not expect our existing findings to be limited by the number of replications.

Thank you very much for your consideration. We look forward to hearing from you.

Sincerely,

Shweta Bansal, PhD
Georgetown University
