# Supplementary material for: Revealing mechanisms of infectious disease spread through empirical contact networks
Source: PLoS Comput Biol. 2021 Dec 20;17(12):e1009604. doi: 10.1371/journal.pcbi.1009604 (PMC8758098; doi:10.1371/journal.pcbi.1009604)
Supplement: Attachment — Submitted filename: INoDS_ response to PLOS comp bio reviews.docx [file pcbi.1009604.s006.docx]

**Reviewer #1:**

*This manuscript presents a method of inferring model parameters for disease spreading on empirical contact networks. It is also capable of testing the robustness of the network representations given data about who is infected at what time. I think it looks like a contribution of practical importance, even though getting the data needed for input is a difficult task. Below are some specific concerns:*

Response: Thank you for this positive review of our work.

*[Line 9–12] "Constructing a complete contact network model requires (i) knowledge about the transmission route(s) of a pathogen, (ii) a sampling of all individuals in a population, and (iii) a sampling of all interactions among the sampled individuals that may lead to infection transfer." First, if you have done (iii), haven't you also done (ii)? Second, why do you need to know (i)? Yes, you would need to know it to infer the parameters of the compartmental model, but not to construct a contact network?*

Response: Thanks for this question. We agree that sampling all interactions is sufficient for sampling all individuals; however it is not necessary (i.e. one can have a full census of all individuals without having all interactions as well). Additionally, we do believe that knowing the transmission route of the pathogen is key to defining which interactions are relevant for disease transmission (e.g. if a sexually transmitted pathogen is of interest, then the contact network should only be defined based on sexual contacts. For these reasons, we’ve left this sentence unedited.

*[Line 61–65] You say you can use a "dynamic contact network." Above, you defined a contact network as "individuals are represented as nodes, and an edge between two nodes represents an interaction that has the potential to transmit infection". Exactly how does dynamism enter this definition?*

Response: Thank you for this comment. To clarify the dynamic terminology, we have added “A dynamic contact network model tracks interactions evolving over time due to social, demographic or environmental processes as well as perturbations [Bansal et al, 2010].” to the revised manuscript.

*[Line 62–63] If you have a temporal network version about the SIS or SIR model, it is a two-parameter model. You cannot reduce parameters and omit the recovery rate as in static network epidemiology. Thus, your tool also needs to estimate the recovery rate (or disease duration, depending on your model of infection duration).*

Response: We agree that estimating the recovery rate parameter for SIS or SIR model is essential, which our model does estimate. We now clarify this point in the Introduction and “Parameter estimation and data augmentation of infection timings” subsection of Materials and Methods as such:

Introduction:

“The tool can estimate the per-contact transmission rate and recovery rate for various types of infectious diseases (SIS and SIR) and can be easily extended to incorporate other complex models of disease spread. “

Materials and Methods:

“For infections that follow a SIS or SIR disease model, it is also essential to impute the recovery time of infected individuals for accurate estimation of infected degree. To do so, we adopt a similar data augmentation approach as described to sample from the set of possible recovery time-points.”

*[Line 63] In the light of the previous question, you can omit the SI model since it is contained in the SIS and SIR models.*

Response: An SI model is indeed distinct from its SIS counterpart in dynamics so we have left this sentence unchanged for completeness.

*[Fig. 1] The smallest text is not readable.*

Response: We have increased the font size of the text in Figure 1.

*[Around line 87] How do you randomize the network? If you randomize a dynamical network, there are many options that all destroy different structures of the original data. See Gauvin et al., Randomized reference models for temporal networks https://arxiv.org/abs/1806.04032*

Response: We agree that there are many options of randomizing dynamic networks -- each with its own advantages and drawbacks -- with no established gold-standard approach. In our model, we randomize dynamically by performing double-edge swaps at each time snapshot and setting edge weights to the average edge weight of the original snapshot. Since the impact of edge-weights and higher-order network structure on infectious disease transmission is unclear (and often context-dependent), we believe that preserving the degree-distribution is the most conservative approach of generating null networks. We clarify our randomization procedure in the Results section:

Results:

“Null hypothesis is expressed as a uniform distribution over randomized networks generated by permuting 10% -- 100% edge connections of each time-slice using double-edge swap procedure \citep{Molloy1995} and assigning each edge the average edge weight of the original snapshot.”

*[Around line 87] What is the logic of randomizing different percentages of the links? When you randomize the network, you destroy the particular structure that affects the disease spreading. It is not telling you whether your network is robust to errors because random changes make the network more random, i.e., less like the structured, real networks. If you consider networks obtained by random changes as a model, this is systematically biased toward randomness and should certainly be a bad model.*

Response: By generating networks with different percentages of permuted links, we assume uniform prior distribution over the degree of permutation (ranging from 0 to 100%). This allows us to perform Bayesian null hypothesis testing, where the null hypothesis is compared against an alternative hypothesis (H2) with a prior distribution that puts an infinitely dense spike at 0% permutation. Bayesian inference reallocates credibility across the two hypotheses (H1 vs H2) by reallocating credibility across the various permutation levels (0-100%). Our null formulation does not systematically bias towards randomness because it puts equal probability weight on all levels of permutations.

*[Line 104] " 0.039 ( 0.003 SD)" What is this notation? Do you mean 0.003 times the standard deviation? or that 0.003 is the standard deviation?*

Response: 0.003 is the standard deviation. To clarify, we have edited the statement to:

“For example, we estimated an average $\beta$ value of 0.039 (SD = 0.003) using INoDS for disease simulations involving a simulated pathogen with $\beta$ value of 0.04.”

*[Line 118] What is an "edge" in a dynamic network? Please conform to the temporal-network literarure. Masuda & Holme, Predicting and controlling infectious disease epidemics using temporal networks, F1000Prime Rep. 5, 6 (2013).*

Response: An edge in our dynamic network corresponds to a contact (of duration between 1 and 100 time steps) in the terminology of Masuda and Holme. We have added this to the Methods section, under the Validating INoDs Performance subsection.

*Continuing the previous point. There are papers in the temporal-network epidemiology literature that relate to this work. One such is: Holme & Rocha, Impact of misinformation in temporal network epidemiology, Network Science 7, 52-69 (2019).*

Response: The cited paper by Holme and Rocha is a really interesting one; however, it focuses on the role of erroneous (temporal) network data on the prediction of disease dynamics. The relevant analysis in our study focuses instead on how erroneous network data can impact our prediction of the pertinent contact network.

*[Line 122] How do you define "true synthetic network"?*

Response: We created a dynamic synthetic network to perform model validation, which we refer to as the “true synthetic network” in the results. We clarify this point in the Results section:

“We validated the performance of INoDS using a simulated dataset. This dataset was generated by performing numerical disease simulations on a synthetic dynamic network (henceforth called the true synthetic network) where per-contact transmission rate, $\beta$, ranged from 0.01 to 0.1.”

*[Fig. 3] What is the point of plotting panel A? You could just state what we see in a sentence.*

Response: We kept panel A to be consistent with the 3-panel format of Figure 2, and also to visualize the three steps of model inference described in Figure 1.

*[Fig. 3 caption] If you remove edges not involved in a spreading path, aren't you introducing a bias? Those edges should, by definition, be less important for the epidemics.*

Response: In our sensitivity analysis, we sought to test the hypothesis that INoDS would perform equally well when the missing network data is not involved in the spreading path. We chose this for two reasons (and now highlight this in the main text in the Results section):

“We focus on this type of missing data as infected individuals and their contact are more likely to be observed (particularly for infections with observable symptoms). Methodologically, this approach also allows us to tease apart INoDS's performance when both the complete and incomplete networks have equal ability to explain the propagation of disease. If robust to missing data, we expected INoDS to recover the same parameter estimates and model evidence as the true and complete synthetic network.”

By contrast, when the missing data is involved in the epidemic path, we do expect INoDS performance to be lower because the input data is missing a fundamental piece of information. To clarify this difference, we have included an additional analysis testing the INoDS performance on random missing data in the supplement, and explain in the text as follows:

“In the supplement, we tested INoDS performance with random missing data (Figure S3). We found that the deviation of estimated $\beta$ increases with the degree of missing data, and networks with missing data had lower evidence compared with the true synthetic network. This is because removing nodes/edges involved in the transmission process lowers the ability of network hypothesis in explaining the propagation of disease outbreak. Additionally, removing cases (i.e., infected status from nodes) from infection time-series results in lower quality infection data compared to data where all infection events are documented.”

**Reviewer #2:**

*Sah, et al. present an exciting technique that is leaps and bounds beyond of the "comparable" approaches they test it against in the study of contact network relevance to disease spread. This work has great potential to address some of the most pressing concerns in disease ecology, namely a rigorous way to assess and compare contact networks as explanatory tools in the spread of infectious disease.*

Response: We thank the reviewer for their positive review of our work.

*Despite my excitement and generally positive impression, I have one main concern and two secondary ones that I outline below.*

*# Validation with respect to missing data:*

*The authors perform three main tests to validate their approach in the face of missing data: they remove nodes, edges, or cases and then run the model to see if it still performs well in each of its three tasks: estimating parameters, determining that the network is relevant to disease spread, and performing model selection between the "true network" and the pruned ones.*

*My main issue is that under the current methods, the authors only consider nodes and edges that are not involved in the simulated disease transmission for removal. Besides being unrealistic from a data-scarcity perspective (we rarely know whether the individuals we have missed were involved in the transmission chain or not), this additionally (I would argue undesirably) alters the model-perceived disease prevalence.*

*Because (by definition) none of the removed edges were involved in the transmission process, many (most?) of them were connecting two susceptible nodes and thus wouldn't have come into the likelihood calculation anyway. The removal of edges should functionally increase the perception of beta, since edges that remain are more likely to result in further infections, but this is not what we see in Figure 3 -- I wonder if the authors have an intuition for why their model continues to perform so well*

**Response:** We thank the reviewer for this insightful comment. Our sensitivity analysis does not alter the model-perceived prevalence because the likelihood calculation depends only on the neighboring nodes of the focal node that are infected at the previous time-step (equation 1). Removal of edges not involved in the transmission process, therefore, does not alter the estimation of beta because the model does not take these node/edge connections into consideration while estimating beta.

To further clarify the process, we’ve added a supplementary analysis to the manuscript that demonstrates the impact of removing nodes/edges at random rather than selectively. As the reviewer correctly states, when nodes/edges are randomly removed, increasing missing data causes an increase in the deviation of the \beta parameter (a new Figure S2). We clarify this point in the results as follows:

“In the supplement, we tested the performance of INoDS when data is missing at random (Figure S2). We found that the deviation of estimated $\beta$ increases with the degree of missing data, and networks with missing data had lower evidence compared with the true synthetic network. This is because removing nodes/edges involved in the transmission process lowers the network’s ability to explain the propagation of disease outbreak. Additionally, removing cases (i.e., infected status from nodes) from infection time-series results in lower quality infection data compared to data where all infection events are documented.”

*The removal of nodes is not really a distinct treatment from the removal of edges, as when nodes are removed, the edges connected to them are removed as well. We would thus expect nodes to have (at least) as big of an effect as just removing edges. Importantly, this removal also increases the prevalence of disease within the network (because we are only removing healthy nodes). This combined with the above is likely why we see an increase in epsilon, though the lack of a response in beta is surprising, as before.*

Response: We agree that the removal of nodes is not a distinct treatment from removal of edges. However, removal of nodes/edges not involved in the transmission tree does not affect the beta estimation as the likelihood calculation in our model only considers neighboring nodes that were infected in the previous time step (equation 1). The epsilon estimates, therefore, are close to zero even with increasing percentages of nodes/edges removal (see figure below).

**Figure S2:** Estimate of epsilon with increasing percentages of nodes/edges removal that were not involved in infection spread path.

*Finally, removing cases is the only change that alters the actual infection data, but in this case, nodes are not removed but rather their infectious status is never recognized (assuming they follow the procedure from VanderWaal, et al. (2016)). This seems like it would have the biggest effect on beta, but those results are not presented in this work. It does yield the smallest absolute epsilon, but might have something to do with effectively reducing prevalence in the population. Thus, less extrinsic forcing is needed to explain the data, even in the absence of beta (more on this below).*

**Response:** Thanks to this comment, we have added results for when case data is removed at random (Figure S3), following the case removal procedure from VanderWaal (2016). We found that removing cases has the least effect on beta and epsilon estimation, compared with random removal of nodes or edges. By contrast, random removal of cases has a higher effect on beta and epsilon estimation compared to removal of nodes/edges not in the infection path. .

*I would like to see two additional pruning procedures: removing nodes truly at random (infected or not), and the same for edges. This would keep the prevalence approximately equal and would be a more realistic scenario. I expect these will have a larger effect on beta, since we are losing information about the actual spread of disease.*

Response: We thank the reviewer for this suggestion, and as we state above, we’ve now included this supplementary analysis in the manuscript.

*It might be interesting to connect the neighbors of removed nodes via the addition of edges, since there is still a path for infection even if we don't observe it, but this might be beyond the scope of this work.*

**Response:** This certainly is an interesting point which we plan to explore in future work.

*In Figure S2, the authors consider the absolute deviation of epsilon from 0, but I am not convinced this is the most informative way to present the model's reliance on unobserved transmission. Given a timeseries and a network without edges, there is some best-fit (i.e. maximizing the likelihood function in equation 2) epsilon for regenerating the data. Any transmission events that can be better explained by a network edge (and therefore beta parameter) reduce the model's expectation for epsilon. I am curious how a figure like S2 would look if the y-axis were the relative error (to this maximum) rather than absolute error (from 0).*

**Response:** Thank you for this insightful comment. As suggested, we have replaced the figure (now Figure S3) to show deviation of epsilon from the expectation when beta=0. We have also added text in the Discussion clarifying these results:

“In principle, the background transmission rate parameter, $\epsilon$, in INoDS is similar to the asocial learning rate used in the network based diffusion analysis approach in the behavior learning literature \citep{Franz2009, Aplin2013}. The background transmission parameter in our model serves to approximately assess transmission which is unexplained by the edge connections of the network hypothesis. Under the scenario of a network hypothesis with no edges, the best fit $\epsilon$ would indicate that all disease data is generated by unobserved transmission. Any transmission events that can be better explained by a network edge reduce the model's expectation from this maximum $\epsilon$ estimate. Relative deviation of $\epsilon$ from the maximum possible value is therefore highest for a network hypothesis with no missing data. The relative deviation of $\epsilon$ declines with increasing missing data, although it is more sensitive to missing network information compared with missing information in infection time-series data (Figure S3). “

*Finally, my understanding is that the time of infection is assigned (uniformly) randomly between the two timepoints of an individual being susceptible and infectious. It seems a reasonable assumption that this distribution is non-uniform insofar as the likelihood of infection will depend on the node's degree as it changes throughout this window.*

**Response:** The reviewer is correct in their assumption that the likelihood of infection depends on the number of neighboring nodes that are infected, which our model does take into account while estimating the infection timing of infected nodes when only diagnosis data is available.

*# Comparison to previous methods:*

*One of the variables noted to be important to success of the approach of VanderWaal, et al. (2016) is pathogen prevalence. Did the authors detect a similar dependence in their method?*

**Response:** Our method is robust to a wide range of pathogen prevalence, with the exception of prevalence below 10%. For example, in Figure 4, the prevalence level ranged from 8.1% (corresponding to beta = 0.01) to 99.8% (corresponding to beta=0.1). We found that INoDs accurately established epidemiological relevance across all ranges of prevalence when no network or disease data was missing considering a Bayes Factor (BF) threshold of 0.5. At a higher threshold of BF =3, the power of INoDS at established epidemiological relevance was low for beta=0.01. We now clarify this point in the results section:

“To do so, we performed simulations of infection with $\beta$ ranging from 0.01 to 0.1, corresponding to disease prevalence of 8.1\% to 99.8\%, respectively.

“We found that INoDS accurately established epidemiological relevance across a wide range of $\beta$ when no network or disease data was missing. At $\beta$=0.01, however, the power of the model is lower compared to k-test (Figure 4). For values of $beta$ beyond 0.01, the performance of INoDS in establishing epidemiological relevance surpasses two previous approaches -- the k-test procedure and the network position test. The power of the k-test in detecting epidemiological relevance of an observed contact network decreases with an increasing amount of missing data and transmission rate of pathogen. Of the three approaches, the network position test has the lowest power in detecting epidemiological relevance. ”

*Along these lines, I am having trouble reconciling Figure 4 with Figure 3 from VanderWaal, et al. (2016) -- how do the authors find nearly 0 power under (presumably) similar parameterizations that VanderWaal, et al. find nearly 1 (i.e. beta = 0.04 and 0.133)?*

**Response:**  We found that the k-test performed reasonably well for low to moderate disease prevalence levels (corresponding to beta 0.01 to 0.03 in our simulations), which corroborates Figure 3 from *VanderWaal, et al. (2016).* Higher levels of beta in our simulations correspond to high disease prevalence. At high disease prevalence, permuted distribution of k-statistics yield similar value as the observed k-statistic because most nodes in the networks are infected, resulting in lower power of the test. We have added a figure summarizing disease prevalence in our simulations in the Supplement:

Figure S5: Disease prevalence corresponding to different values of beta used in our simulations for Figure 4.

*378 -- the authors use the Kass & Rafferty (1995) interpretations of BF (i.e. > 0.5 is "signficant"), but this seemed to be the most generous of interpretations that I've encountered. In most cases, this doesn't matter, as the authors report the BF directly, however in Figure 3, I wonder how much the left column would differ if 2, 3, or 10 (sensu Lee and Wagenmakers (2014)) were used instead.*

**Response:**  We agree with the reviewer that a threshold of Bayes factor and p-value deemed “significant” varies widely with the literature, and is often context dependent. Our goal was to perform a fair comparison between three methods by choosing a Bayes Factor that would closely correspond to the p-value of 0.05 used in the other two approaches. We updated our analysis to use the most recent recommendation of BF upper bound of 2.44 (Benjamin and Berger, 2019). We have added results of this analysis in the text:

“We found that INoDS accurately established epidemiological relevance across a wide range of $\beta$ when no network or disease data was missing. At $\beta$=0.01, however, the power of the model is lower compared to k-test (Figure 4). For values of $beta$ beyond 0.01, the performance of INoDS in establishing epidemiological relevance surpasses two previous approaches -- the k-test procedure and the network position test. The power of the k-test in detecting epidemiological relevance of an observed contact network decreases with an increasing amount of missing data and transmission rate of pathogen. Of the three approaches, the network position test has the lowest power in detecting epidemiological relevance. ”

*# Code/reproducibility:*

*The link provided in the text (https://bansallab.github.io/INoDS-model/) leads to a page that does not contain a link to download the software, requiring the reader to have sufficient knowledge of github to navigate to https://github.com/bansallab/INoDS-model/ before gaining access.*

**Response:** We have corrected the link to ”<https://github.com/bansallab/INoDS-model>” which provides a link to download the software.

*It would additionally greatly improve accessibility if some sample data were provided in the repository such that a user can get it running right out-of-the-box, so to speak. As it is, even the referenced datasets are not readily available. I was unable to find either dataset within the Harvard Dataverse (as referenced in the text), and could find no record of the bee dataset elsewhere either. I did find the lizard dataset in DataDryad (https://datadryad.org/stash/dataset/doi:10.5061/dryad.jk87h), but the data format in this repository is not easily converted into one acceptable for INoDS.*

**Response:** The empirical datasets are available at Harvard Dataverse (<https://doi.org/10.7910/DVN/YAHRDJ>). We have added the link in the Materials and Methods section.

“

Empirical datasets used in this study are available at https://doi.org/10.7910/DVN/YAHRDJ. ”

*I don't think the randomization code used in this work was provided -- does your particular configuration model algorithm allow non-simple graphs? If so, I would recommend re-running with one that does not in order to make the random ensemble representative of empirically obtainable data.*

**Response:** Our randomization procedure indeed only allows simple graphs (i.e. graphs without self-loops and multiple edges). The randomization code follows a double-edge swap procedure and can be found here: <https://github.com/bansallab/INoDS-model/blob/master/INoDS_convenience_functions.py>.

*# Other Minor Points*

*My read of Eq. 2 is that it this formulation is particular to a disease model without re-infection -- if so, this should be stated explicitly*

**Response:**  We have added this point in the Discussion as follows:

“As with all models, the results from INoDS should be considered within the context of model assumptions. First, we assumed that the infection process has no latent period. In the future, disease latency can be incorporated into the model by using a data-augmentation technique, similar to what we use for inferring infection times. Second, we assumed that the infectiousness of infected hosts and susceptibility of naive hosts is equal for all individuals in the population. Heterogeneity in infectiousness of infected hosts and the susceptibility of naive hosts can be incorporated as random effects in the model. Third, in the current version of our model, we do not consider re-infection of hosts. Re-infectivity of hosts can be easily incorporated by allowing multiple time-points of infection acquisition in equation (2).”

*l 136-138 -- I'm not sure I understand why this was "expected"*

**Response:** In this test of model robustness, we deleted only those nodes/edges that were not a part of the path of simulated infection spread. If robust to missing data, we expected INoDS to estimate the same model evidence for the true, complete synthetic network and the incomplete network. Our results confirmed our expectation because the true synthetic network, in fact, did not have a higher evidence compared with incompletely sampled networks. We clarify this point in the “Robustness to missing network data” subsection of the results:

“Next, we tested the robustness of INoDS against two potential sources of error in network data collection: incomplete sampling of individuals in a population (missing nodes) and incomplete sampling of interactions between individuals (missing edges). To create networks with missing data, we randomly deleted 25--75% of nodes and edges from the true synthetic network that were not a part of the path of simulated infection spread. This approach allowed us to tease apart INoDS's performance when both the complete and incomplete networks have equal ability to explain the propagation of a disease outbreak. If robust to missing data, we expected INoDS to recover the same parameter estimates and model evidence as the true and complete synthetic network.”

“ As expected, log Bayes factor was less than 2 for all degrees of missing data indicating that the true synthetic network does not have higher evidence compared with incompletely sampled networks. Together, we found that the performance of INoDS was unaffected by missing network data if the missing nodes/edges are not a part of the outbreak path.”

*l 157 -- this reference should be to figure 4, I believe*

**Response:** We have corrected the reference as figure 4.

*l 362 -- should this section heading have an "epsilon" in it?*

**Response:** We have edited the section heading to include “epsilon”.

*l 411 -- do I understand these methods correctly that there was only one simulation per missing data scenario-beta combination? If so, I assume the paired outliers in Figure 3c are thus corresponding to particular beta values. I highly recommend performing greater replication in this analysis.*

**Response:** We performed two simulations per missing data scenario-beta combination, so each box in Figure 3 represents a total of 20 simulations.

*## Figure 2*

*Contrary to the caption, the boxplot whiskers do not encompass the range (as evidenced by the presence of points outside of these ranges). More likely:*

*"The upper whisker extends from the hinge to the largest value no further than 1.5 * IQR from the hinge (where IQR is the inter-quartile range, or distance between the first and third quartiles). The lower whisker extends from the hinge to the smallest value at most 1.5 * IQR of the hinge. Data beyond the end of the whiskers are called "outlying" points and are plotted individually." -ggplot2 geom_histogram help page*

*This description is also present in other captions.*

**Response:** Thank you for this comment. We have corrected the caption throughout the main text and supplement as follows:

“The middle black line in each box plot is the median, the boxed area extends from the 25th to 75th quartile, and whiskers extended from the hinge to the largest/smallest value no further than 1.5 times the inter-quartile range.”

*a) Plotting the beta/epsilon values directly here makes it somewhat difficult to gauge any deviation of the mean estimate from the ground truth, or any trends in deviation along beta*. Perhaps switch the vertical axis to show the absolute error or add a 1:1 line for reference. I slightly prefer the former approach, since that would allow a better understanding of any change in epsilon with increasing beta as well, which is hard to note in the current figure due to the difference in scales.*

**Response:** Following this suggestion we have plotted absolute error along the y-axis for Figure 2a. We have edited the subfigure caption as follows:

“(a) **Step 1**:Absolute error in estimates of per-contact transmission rate parameter β (orange circles) and background transmission rate ε (purple circles) for the simulated dataset with disease transmission rate (β*) ranging from 0.01 to 0.1. The true value of background transmission rate (ε*) is zero. The filled black circle indicates the average absolute error and the error bars indicate standard deviation around the mean value.”

*b) Do these boxplots aggregate log BF across networks that vary in their degree of permutation (i.e. combining what is subdivided in panel c)?*

**Response:** For panel a and b, no network permutation is performed. These boxplots represent the true and original synthetic network on which disease simulations were carried out.

*## Figure 3*

*I wasn't sure why omitted cases is not presented here, despite being in Figure 4 and in the SI*

**Response:** The missing data analysis performed in Figure 3 is different from Figure 4 and the figure in the SI because the former evaluates removal of data not involved in infection spread pathway while the latter evaluates random removal of data. Removal of cases, while possible when removing data randomly (Figure 4 and SI Figure), is not possible when the criteria is to remove data not involved in infection spread (because by definition, an infection acquisition event *is* a part of infection spread pathway).

*a) Are the differences from expectation actually 0 or is this a vertical-axis scaling issue? and is this absolute error? if so, I would recommend relative error here, since beta spans an order of magnitude.*

**Response:** Following this suggestion, we have changed the y-axis to relative error and reduced the scale of the vertical axis. The differences from the expectation are indeed close to zero.

*## Figure 4*

*caption -- "Statistical power of INoDS was ," [delete was]*

**Response:** We have removed “was” from the caption.

*## Figure 6*

*Is there a missing network in this figure? I expected 5 networks given biweekly sampling for 70 days.*

**Response:** The lizards were fitted with GPS loggers and their locations were recorded every 10 minutes. The networks were constructed by aggregating interactions (based on physical proximity) over 24 hours for 70 continuous days, yielding 70 static network snapshots. For clarity, we present only 4 out of the 70 network snapshots. We clarify this point in the caption:

“For ease in visualization, four networks summarizing interactions at day 15, 30, 57 and 70 are shown out of a total of 70 static network snapshots.”

*I'm a little confused by the table here -- shouldn't frequency weighted at site 2 have an asterisk after it? and why does it have "frequency" in parentheses? Finally, am I reading this correctly insofar as the lower CI bound for the beta and epsilon of the site 1 binary network are equal to the estimates themselves (0.017 and 0.046, respectively)?*

**Response:** Thank you for the careful observations. We have added the missing asterisk and deleted “(frequency)”. The reviewer is correct about their observation of lower CI bound for beta and epsilon of site 1 binary network. While the lower bounds are slightly lower (0.01671 and 0.04594) than the estimates, rounding these to three decimal points makes these values equal to the estimates.

**Reviewer #3:**

*This is an interesting and well written paper describing the development of a tool, INoDS (Identifying contact Networks of infectious Disease Spread), that can be used to perform network model selection and establishes the statistical significance of a contact network model.*

*General comments:*

*This work is very timely. The introduction, methods, and results are well described and easy to follow.*

Response: Thank you for the positive feedback.

*However, there are few key components in the methods and results that need more explanation. Something that the authors need to explain in more detail is why the bumble bee and lizard case studies were chosen and why there was a need to examine both and not just one, for example.*

Response: Following this suggestion, we have added details in the Results section regarding our choice of empirical datasets as follows:

“We chose these two empirical datasets because they represented two distinct (i) host taxonomic class, (ii) models of disease spread (SI vs SIS), and (iii) disease data collection methodology (infection timing known for bumble bee dataset vs unknown for sleepy lizard dataset, and (iv) type of network data (fully connected in bumble bees vs sparsely connected in sleepy lizards). “

*Additionally, while the introduction, methods, and results were well developed, the discussion is currently a bit weak, as it essentially re-iterates much of the information given in the abstract, introduction, and results.*

*The authors do not discuss their work in the context of other studies (or do so at a very shallow level), there is no discussion of limitations nor of future directions and next steps. I would suggest cutting much of the information that repeats the introduction (e.g., why this tool is needed) and results, and provide a deeper discussion of how this tool should be used, which systems might need it most, and what the limitations are.*

Response: We have restructured our Discussion section, adding context with respect to previous studies, adding a limitation section and future directions.

*Finally, the authors need to check some of their references and figure citations in the main text before publication as there are errors (e.g., some figures are incorrectly referenced in the main text (e.g., figure 3 instead of 4) and figure 6 was not referred to at all in the main text).*

Response: We have corrected the referencing of figures in the manuscript.

*Specific comments:*

*Line 63-64: provide examples in parentheses of the type of complex models*

Response: We have taken this suggestion and revised the text as:

“The tool can estimate the per-contact transmission rate and recovery rate for various disease progressions (e.g. SI, SIS and SIR) and can be extended to incorporate complexities in transmission (e.g. individual-level heterogeneity in susceptibility; latent period following infection).”

*Line 94-95: More detail on why those two empirical datasets were chosen, and why the need to look at both. This can be stated either here or in the methods.*

Response: As mentioned above, we have added details on our dataset choice to the Results section as follows:

“We chose these two empirical datasets because they represented two distinct (i) host taxonomic class, (ii) models of disease spread (SI vs SIS), and (iii) disease data collection methodology (infection timing known for bumble bee dataset vs unknown for sleepy lizard dataset, and (iv) type of network data (fully connected in bumble bees vs sparsely connected in sleepy lizards). “

*Line 157: I believe the authors are referring to figure 4 here not figure 3.*

Response: We have corrected the reference to figure 4. Thank you.

*Line 292-293: In what way? Perhaps give examples.*

Response: Following this suggestion, we have added an example as follows:

“Since data-collection for network analysis can be labor-intensive and time-consuming, our approach can be used to make essential decisions on how limited data collection resources should be deployed. For example, under a limited capability of recording real-time interactions between hosts, INoDS can identify the minimum time-resolution required during data collection for a network model with sufficient statistical ability to establish epidemiological relevance.”

*Line 458-459: This needs to be stated earlier in the manuscript as it helps understand why this dataset was used as an example.*

Response: We have added these lines to the Results section to clarify our choice of the two empirical datasets:

“We chose these two empirical datasets because they represented two distinct (i) host's taxonomic class, (ii) models of disease spread (SI vs SIS), and (iii) disease data collection methodology (infection timing known for bumble bee dataset vs unknown for sleepy lizard dataset, and (iv) network connectivity (fully connected in bumble bees vs sparsely connected in sleepy lizards).”

*Figures:*

*Figure 1 – In the first inferential step, need to define k. Since the methods are at the end of the manuscript, it would be good to define it here as well. In the third inferential step, might be good to explain that a Bayes factor above 3 provides support for one hypothesis over the other (for those less familiar with Bayesian statistics).*

Response: We have edited the Figure 1 caption to define k and explain the significance of the Bayes Factor:

“A schematic of our algorithm. **Observed data**(left panel): INoDS utilizes an observed infection time-series data to estimate statistical evidence towards a static or dynamic contact network hypothesis (or hypotheses) using a three-step procedure. Shown here is an example of two competing network hypotheses based on behaviors A and B that potentially cause infection transfer. **Inferential steps** (right panel): In the first step, the tool estimates per-contact transmission rate parameter 𝛽, and background transmission rate parameter ε which captures the components of infection propagation unexplained by the edge connections of the network hypothesis. Here, the total infected connections of the focal node *i* (*k_i_*) is 2. Second, to estimate the epidemiological relevance of the network hypothesis, Bayesian hypothesis testing is performed. The prior distribution shows that the null hypothesis (\textit{M}=1) assumes a uniform distribution over randomized networks generated by permuting 10% -- 100% of edge connections in the contact network (H_A_), whereas the alternate hypothesis (*M*=2) is a spike-shaped distribution such that only the contact network (H_A_, 0% permutation) has non-zero probability. The distribution on model index shifts to M=2 if the alternate hypothesis has higher posterior probability than the null. Third, model selection of competing network hypotheses is performed using Bayes Factor (BF). A Bayes factor above 2.44 is considered to be decisive support for one hypothesis over the other.”

*Figure 3 – panels a-c are not listed in the figure but are when the figure is referenced in the text.*

Response: We have added subfigure labels a-c in Figure 3

*Figure 6 – not referred to in main text*

Response: We have added a reference to Figure 6 in the Results subsection “Identifying transmission mechanisms with imperfect disease data: case study of *Salmonella enterica* Australian sleepy lizards”

*Figure 6 – suggest adding days in parentheses for the time on the x-axis.*

Response: We have followed this suggestion and modified the x-axis as Time (days).
